# Association between Polyphenol Intake and Hypertension in Adults and Older Adults: A Population-Based Study in Brazil

**DOI:** 10.1371/journal.pone.0165791

**Published:** 2016-10-28

**Authors:** Andreia Machado Miranda, Josiane Steluti, Regina Mara Fisberg, Dirce Maria Marchioni

**Affiliations:** Department of Nutrition, School of Public Health, University of Sao Paulo, Sao Paulo, SP, Brazil; UMR INSERM U866, FRANCE

## Abstract

**Background/Objective:**

Hypertension is an important risk factor for cardiovascular disease, and diet has been identified as a modifiable factor for preventing and controlling hypertension. Besides, epidemiological studies have suggested an inverse association between polyphenol intake and cardiovascular diseases. The aim of this study was to evaluate the association between the intake of polyphenols and hypertension in a general population of Sao Paulo.

**Methods:**

Data came from the ‘Health Survey of Sao Paulo (ISA-Capital)’ among 550 adults and older adults in Sao Paulo, Brazil. Diet was assessed by two 24-hour dietary recalls (24HR). Usual intakes were calculated using the Multiple Source Method. Polyphenol intake was calculated by matching food consumption data from the 24HR with the Phenol-Explorer database. The associations between the hypertension and tertiles of the total and classes of polyphenols intake were tested by multivariate logistic regression analysis.

**Results:**

After multivariate adjustment for potential confounding factors the findings showed an inverse and linearly association between the hypertension and highest tertiles of tyrosols (OR = 0.33; 95%CI 0.18, 0.64), alkylphenols (OR = 0.45; 95%CI 0.23, 0.87), lignans (OR = 0.49; 95%CI 0.25, 0.98), as well as stilbenes (OR = 0.60; 95%CI 0.36, 0.98), and other polyphenols (OR = 0.33; 95%CI 0.14, 0.74). However, total polyphenol intake, and phenolic acids were significantly associated only in the middle tertile with hypertension and flavonoids were not significant associated.

**Conclusion:**

There is an inverse and linearly association between the highest tertile of some classes of polyphenols, such as, tyrosols, alkylphenols, lignans, stilbenes, other polyphenols and hypertension.

## Introduction

Polyphenols are considered to have beneficial effects on human health and provide protection against several chronic diseases, such as cardiovascular diseases (CVD) [1, 2]. They are common constituents of the human diet, present in plant-derived foods and beverages, e.g., fruits, vegetables, nuts, seeds, herbs, spices, cocoa, tea, coffee, wine, and they represent more than 8000 phenolic structures [3, 4]. Dietary polyphenols are divided into four main classes: flavonoids, phenolic acids, stilbenes, and lignans, that are largely present in a glycosidic form (glycosides of flavonoids, lignans, and stilbenes) or as esters (phenolic acids esterified to polyols such as quinic acid) [5].

Dietary phenolic compounds have a protective role against cardiovascular risk due to the numerous chemical and structural properties, and biological effects including high antioxidant capacity *in vitro* and *in vivo*, anti-inflammatory and anti-hypertensive effects, and improved endothelial function [6, 7], produced by different mechanisms and, in some cases, different compounds [2]. Thus, a plausible hypothesis is that the polyphenols can stimulate the formation of vasoprotective factors, such as nitric oxide (NO) and endothelium-derived hyperpolarizing factor (EDHF) to promote vasodilatation, inhibit platelet aggregation in humans, and they can also improve vascular smooth muscle function, by reducing the excessive vascular oxidative stress of pathological blood vessels associated with many cardiovascular risk factors [2, 6].

Hypertension is a highly prevalent cardiovascular risk factor in Brazil (above 30%) [8], and it is the major global health problem, affecting approximately 1 billion individuals and causing 7.6 million premature deaths, as well as 6% of all causes of disability worldwide [9]. This disease is defined as systolic blood pressure (SBP) greater than 140 mmHg or diastolic blood pressure (DBP) greater than 90 mmHg and/or the use of antihypertensive medication [8, 10].

Despite the therapeutic advances, the number of people with uncontrolled blood pressure is also increasing, mainly as a result of the adoption of unhealthy lifestyle habits [11]. In this context, current evidence strongly supports that diet is a determinant factor for preventing and controlling hypertension [11, 12]. In addition, it is agreed that the diet rich in fruits, vegetables, and low-fat dairy foods and reduced in saturated fat and salt intake, moderated in alcohol intake, and increased potassium intake is inversely associated with high blood pressure (BP) [12, 13].

Furthermore, observational studies have demonstrated an inverse association between consumption of polyphenol-rich foods (e.g. fruits, vegetables, cocoa) or beverages (e.g. wine, especially red wine, grape juice, tea) and the incidence of CVD [14], CVD-related mortality [15] and the risk of overall mortality [16]. However, even now, only one epidemiological study shows the relationship between BP and intake of polyphenols [17].

In this context, the aim of this study was to assess the association of the total polyphenol intake and polyphenol classes (i.e., phenolic acids, flavonoids, alkylphenols, stilbenes, tyrosols, lignans, and other polyphenols) with the prevalence of hypertension.

## Materials and Methods

### Study population and data collection

The study population was selected from the ‘Health Survey of Sao Paulo (ISA-Capital)’, a cross-sectional study of health and living conditions among a representative sample of individuals living in Sao Paulo, south-eastern Brazil, in 2008 and 2009.

A two-stage cluster sampling was used: census tracts and household. In the first stage, the census tracts were drawn using probability of the number of households in the ‘National Household Sample Survey’ (NHSS) conducted in 2005 [18]. In the second stage, the households were drawn using inverse probability of the number of households in each NHSS. The drawing was systematic, and six study domains were defined by age groups and sex: females and males aged 13 to 19 years old (adolescents), 20 to 59 years old (adults) and 60 years old or over (older adults).

A total of 2,691 individuals, aged 12 years old or over, were selected to answer questions about diet, life conditions (e.g. physical activity, and smoking) and socio-demographic information (e.g. age, sex, race, educational level, and family income). Of all the selected participants, 38% (n = 1,029) refused to participate or changed their address/telephone and could not be located or found at home, even after three visits made at different times (during weekdays and weekends). Despite the loss was not differential among census tracts and socio demographic features, sampling weights were recalculated for each individual considering the sample design, the adjustment for non-response, and post stratification adjustment for gender and age group, in order to equalize the socio demographic features of the sample. Of those 1,662 (62%) individuals who participated, 750 subjects (adolescents, adults, and older adults) donated a blood sample, completed two 24-hour dietary recalls (24HR), and anthropometric data as well as arterial BP were measured. For the present study, only adults and older adults were included totalizing a final sample of 550 individuals ‘S1 Fig’.

The study protocol was reviewed and approved by the Ethics Committee at the School of Public Health, University of Sao Paulo (Approval Number: 003.0.162.000–08). A written informed consent form was obtained from all participants included in the current study. We would like to inform that the sample of the present study doesn′t include minors/children and adolescents. However, just to clarify, the main study, ‘Health Survey of Sao Paulo (ISA-Capital)’ also obtained the written informed consent on behalf of the minors/children, according to ethical requirements.

### Assessment of dietary intake

The dietary intake was measured by two multiple-pass 24HR. The first 24HR was administered at households using the Multiple Pass Method [19] and the second 24HR was administered by telephone using the Automated Multiple Pass Method [20]. The telephone calls were made to the participants’ home or their mobile phone. These methods are structured in five steps: 1) a quick list, where participants list all the foods and beverages consumed uninterruptedly; 2) a forgotten list; participants are asked about commonly forgotten foods consumed, such as candies, coffees, and sodas; 3) time and location of food and beverage intake; 4) detailing cycle, that is, a description of the way of preparation and amounts consumed, and 5) final review, that verifies whether a certain food consumed during the day was not previously recorded [19, 20].

The sampling days covered all the days of the week and seasons. Foods reported in each 24HR were critically reviewed to identify any failures in reporting related to the descriptions of the food consumed or to food preparation techniques, including their apportioning and quantification. The dietetic data were entered in the Nutrition Data System for Research software version 2007 (Nutrition Coordinating Center, University of Minnesota, Minneapolis, MN, USA) [21].

The Multiple Source Method (MSM), a statistical modeling technique, was used to estimate the usual dietary intake of polyphenols and nutrients (sodium, saturated fat, fiber and alcohol) and to remove within-person variation. This technique uses two 24HR. First, the MSM calculates the dietary intake of individuals and then builds the population distribution based on individual data [22]. All participants were considered polyphenol consumers in MSM, because the technique could modify the first percentiles of distribution and it does not modify mean of usual intake of polyphenols.

The reporting of implausible dietary energy intake (EI) was estimated using the predicted total energy expenditure (TEE) method [23] and, the standard deviations were calculated using estimates of variation in energy balance components published [24]. In present study, the misreporting of intake was categorized as under-reporting, plausible-reporting or over-reporting.

### Estimation of polyphenol intake and dietary contributors of polyphenols

Data on the polyphenol content in foods were obtained from the Phenol-Explorer database (www.phenol-explorer.eu) that contains data on the content of 502 polyphenols in 452 foods [5].

Polyphenol intake was calculated by matching usual food intake data from the 24HR (using MSM) and the polyphenol content in foods from the Phenol-Explorer database. The individual polyphenol intake from each food was calculated by multiplying the content of each polyphenol by the daily consumption of each food. The total polyphenol intake was calculated as the sum of all individual polyphenol intakes from all food sources reported by the 24HR. Other details on estimation of polyphenol intake are available elsewhere in previous publication [25]. Besides, the total polyphenol intake was categorized according to polyphenol classes, such as phenolic acids, flavonoids, alkylphenols, stilbenes, tyrosols, lignans, and other polyphenols, a wide class which includes alkylmethoxyphenols, furanocoumarins, hydroxybenzaldehydes, hydroxybenzoketones, hydroxycinamaldehydes, hydroxycoumarines, and naphthoquinones, accounted for very low intake (each one <0.1 mg/day).

For dietary contributors of polyphenols, a ratio of the daily total polyphenols provided by the specific food or food group over the total intake of polyphenols from all foods was used to calculate the contribution of each food or food group to the daily total intake of polyphenols [25].

### Anthropometric measures and lifestyle characteristics

A trained research nurse measured body weight and height, using a standardized protocol. Body mass index (BMI) was calculated by dividing weight (kilograms) by the square of height (meters) and then classified as underweight and normal weight (<25 kg/m^2^) or overweight (≥25 kg/m^2^) according to the World Health Organization [26]. The physical activity level was categorized as daily low active or active, according to the international physical activity questionnaire (IPAQ) [27], validated in Brazil [28]. The smoking status was categorized as nonsmoker and former smoker or current smoker.

### Ascertainment of the outcome

The individuals were instructed, by telephone in the day before the visit, to refrain from practicing physical activity 60 to 90 minutes before BP measurement, and do not to eat, drink or smoke within 30 minutes prior to the measurement. During the home visit, BP was measured using an automatic blood pressure monitor (Omron HEM-712C, USA), handled by a nursing technician, who also collected data on medication use, including antihypertensive drugs. BP was measured on the right and left arm, both free of clothes, with the subject in the sitting position and in silence, observing a one-minute interval between measurements, according to the recommendations of the V Brazilian Guidelines on Hypertension for adults [8]. Two other measurements were taken, with the same interval, on the arm that showed the highest BP level. The final BP value was obtained by calculating the simple arithmetic mean of the last two measurements.

Hypertension was defined by using cutoffs according to the national and international recommendations, which classifies hypertension as a SBP≥140 mmHg or a DBP ≥90 mmHg, use of antihypertensive medication, or both [8, 10].

### Statistical Analyses

The baseline characteristics of the participants were presented as medians and interquartile range (IQR) for continuous variables, and frequencies and percentages for categorical variables across tertiles of total polyphenol intake. Differences between tertiles were tested by Kruskall-Wallis test for continuous variables and by the chi-square test for categorical variables. Major food contributors (% contribution to polyphenol class) were also determined.

The associations between the dependent variable (hypertension) and the following independent variables: tertiles of total polyphenol intake, total phenolic acids, flavonoids, lignans, stilbenes, tyrosols, alkylphenols, and others, were tested by multivariate logistic regression analysis to adjust for potential confounders, described below. All covariates with P-value <0.20 in univariate analysis were selected for multiple regression analyses and included in regression model by stepwise forward procedure. Three models were fitted with each independent variable. The first model was adjusted for age (years) and sex (male or female); the second model was further adjusted for race (white or others), educational level (medium-until high school or high-university), BMI (under/normal weight or overweight), smoking (no or yes), and physical activity (low active or active); the third model was additionally adjusted for intake of sodium (milligrams/day), fiber (grams/day), saturated fat (grams/day), alcohol consumption (grams/day), total energy intake (kcal/day), and misreporting of intake (under-reporting, plausible-reporting or over-reporting). The adjusted odds ratio and 95% confidence interval (95% CI) were estimated.

Furthermore, we conducted statistical analysis with the additional adjustment for potassium and calcium intake in the third model, however there was no modifications in measures of association (OR and 95% CI) (unpublished data). Thus, we decided to keep the most parsimonious models, with better adjustment for the Hosmer-Lemeshow goodness-of-fit test.

All analyses were conducted using the appropriate sample weights to account for the complex survey design. Tests for linear trend were calculated by assigning the median value for each exposure category and modeling the values as continuous variables. For all analyses, Stata^®^ statistical software package version 12 was used and a P-value<0.05 was considered statistically significant.

## Results

A total of 550 individuals were available for the final analyses. The sample was comprised of 80.2% adults and 19.8% older adults, 54% women, mostly self-declared white (61.0%), with a university level (69.9%), overweight (56.3%) and non-smokers (58.3%). The mean and median polyphenol intake for the whole population was 392.6 and 360.6 mg/day.

The distributions of potential risk factors for hypertension in this population according to tertiles of total polyphenol intake are shown in Table 1. The highest tertile of total polyphenol intake was significantly associated with age, intakes of saturated fat, dietary fiber and arterial BP. The other characteristics (sex, race, physical activity, educational level, smoking status, BMI, misreporting, sodium and alcohol intake) did not differ significantly across tertiles of total polyphenol intake.

**Table 1:** Sociodemographic, lifestyle and dietary characteristics of participants according to tertiles of total polyphenol intake. ISA-Capital study. Sao Paulo.

	Total polyphenol intake, median (IQR) or n (%)	Tertile of total polyphenol intake, median (IQR) or n (%)			
Characteristics		T1 (Lowest)	T2 (Middle)	T3 (Highest)	P-value ^a^
Number of subjects (N = 550)		167	179	204	
**Sociodemographic**					
Age (years)	43 (31, 56)	41 (29, 53)	40 (32, 57)	47 (33, 59)	0.019^1^
Age group					
Adults	261 (80.2)	88 (84.0)	85 (81.3)	88 (75.1)	0.035^2^
Older adults	289 (19.8)	79 (16.0)	94 (18.7)	116 (24.9)	
Sex					
Male	204 (46.0)	61 (43.2)	66 (49.5)	77 (45.2)	0.593^2^
Female	346 (54.0)	106 (56.8)	113 (50.5)	127 (54.8)	
Race					
White	340 (61.0)	95 (62.8)	111 (56.7)	134 (63.4)	0.549^2^
Others	210 (39.0)	72 (37.2)	68 (43.3)	70 (36.6)	
Educational level					
Medium	287 (30.1)	87 (28.8)	91 (28.9)	109 (32.6)	0.789^2^
High	263 (69.9)	80 (71.2)	88 (71.1)	95 (67.4)	
Body mass index (Kg/m^2^)					
Under and normal weight	194 (43.7)	62 (46.4)	68 (48.2)	64 (36.6)	0.208^2^
Overweight	356 (56.3)	105 (53.6)	111 (51.8)	140 (63.4)	
Physical activity					
Low active	277 (41.5)	77 (37.2)	102 (50.4)	98 (36.9)	0.089^2^
Active	272 (58.5)	90 (62.8)	76 (49.6)	106 (63.1)	
Smoking status					
No smoker	303 (58.3)	89 (61.3)	103 (60.0)	111 (53.6)	0.458^2^
Current smoker	247 (41.7)	78 (38.7)	76 (40.0)	93 (46.4)	
Hypertension					
No	283 (67.9)	82 (62.6)	102 (77.3)	99 (63.9)	0.040^2^
Yes	267 (32.1)	85(37.4)	77 (22.7)	105 (36.1)	
Misreporting					
Under-reporting	94 (17.0)	39 (24.2)	29 (12.9)	26 (13.8)	0.202^2^
Plausible-reporting	349 (66.3)	103 (62.7)	112 (66.7)	134 (69.6)	
Over-reporting	80 (16.7)	15 (13.1)	30 (20.4)	35 (16.6)	
**Dietary**					
Polyphenol intake (mg/d)	360.6 (233.0, 478.6)	192.2 (122.4, 233.0)	360.7 (329.6, 401.9)	566.9 (478.6, 659.7)	<0.001^1^
Alcohol intake (g/d)	0.1 (0.0, 3.7)	0.1 (0.0, 3.7)	0.2 (0.0, 2.8)	0.3 (0.0, 3.7)	0.190^1^
Sodium (mg/d)	3103.3 (2563.4, 3846.2)	2937.2 (2509.9, 3820.7)	3241.8 (2528.2, 3890.1)	3046.4 (2611.0, 3450.9)	0.503^1^
Dietary fiber (g/d)	16.5 (12.9, 19.7)	14.4 (11.8, 18.9)	16.8 (13.8, 19.2)	17.2 (14.2, 20.8)	0.012^1^
Saturated fat (g/d)	17.7 (13.3, 23.5)	18.5 (14.9, 24.8)	17.5 (13.5, 23.5)	17.3 (12.2, 21.7)	0.045^1^
Total energy intake (kcal/d)	1693.2 (1361.1, 2017.5)	1623.1 (1310.9, 2054.5)	1743.4 (1423.9, 2088.6)	1715.3 (1375.4, 2017.5)	0.442^1^

Abbreviation: IQR: Interquartile Range. Comparisons across categories were performed by using ^1^ Kruskall-Wallis test; ^2^ chi-squared. The sample weight was considered for statistical analysis. For this reason, the values of proportion of each category of variable aren′t compatible with the frequency of individuals.

^a^ p-value<0.05 was considered statistically significant.

### Classes of polyphenols and its main food contributors

The main polyphenols′classes were: phenolic acids 290.3 (133.0, 400.3) mg/day, representing 79.9% of total intake of polyphenols, and flavonoids 34.5 (17.4, 61.6) mg/day, representing 11.6% of total intake of polyphenols, whereas other polyphenols, such as, alkylphenols, stilbenes, tyrosols, lignans accounted for lower proportions. The remaining polyphenols were grouped into a wide class of other polyphenols, representing 1.4% of the total polyphenol intake.

The main food contributors according to the different classes of polyphenols are shown in Table 2. The main dietary sources for the total polyphenols were coffee (70.5%) and fruits, especially orange. Also, coffee was the primary food item contributing to phenolic acids intake (92.3%). Major contributors of intake of flavonoids were orange (15.4%), orange juice and beans. Tyrosols were derived from olives (56.0%) and olive oil; lignans were derived from sesame seed oil (71.3%) and nuts; alkylphenols were present in whole grain bread (66.8%) and whole grain flour; stilbenes in red wine (75.8%) and grapes; whereas other polyphenols came mostly from coffee (75.0%), orange and lemon juices.

**Table 2:** The main food contributors of the polyphenols intake according to classes of polyphenols. ISA-Capital study. Sao Paulo.

		Main food contributors (%) ^1^					
		Rank					
	Median (IQR), mg/d	1^st^	2^nd^	3^rd^	4^th^	5^th^	6^th^
**Total polyphenols**	360.6 (233.0,478.6)	coffee (70.5)	orange (2.2)	tangerine (2.1)	orange juice (2.1)	potatoes (2.0)	beans (1.9)
**Phenolic acids**	290.3 (133.0, 400.3)	coffee (92.3)	potato (2.6)	beer (0.9)	apple (0.6)	tomato (0.4)	refined flour (0.3)
**Flavonoids**	34.5 (17.4, 61.6)	orange (15.4)	orange juice (13.9)	beans (13.2)	onion (8.2)	grapes (6.6)	apple (6.4)
**Tyrosols**	1.8 (1.0, 3.2)	olives (56.0)	olive oil (30.2)	beer (9.9)	red wine (2.4)	vinegar (1.3)	white wine (0.1)
**Alkylphenols**	1.0 (0.5, 1.7)	whole grain bread (66.8)	whole grain flour (22.1)	refined flour (6.0)	breakfast cereals (4.7)	beer (0.4)	--- (0.0)
**Lignans**	0.1 (0.1, 0.2)	sesame seed oil (71.3)	nuts (19.7)	sesame seeds (3.9)	olive oil (2.0)	flaxseed (1.7)	bread refined flour (0.7)
**Stilbenes**	<0.1 (<0.1)	red wine (75.8)	grape (11.1)	strawberry (7.3)	white wine (4.6)	dark chocolate (0.5)	lentils (0.3)
**Others**	1.7 (0.9, 2.3)	coffee (75.0)	orange juice (21.5)	lemon juice (2.3)	olives (0.4)	beer (0.3)	olive oil (0.2)

---: no food contributor

^1^ In the rank, the number between parentheses refers to the percentage of total intake within each polyphenol class.

### Total polyphenols intake, polyphenol classes and hypertension

Table 3 presents the associations between hypertension and the tertile of intake of total polyphenols and their main classes.

**Table 3:** Association between tertiles of polyphenol intake (total and main classes) and hypertension. ISA-Capital study. Sao Paulo.

	Tertile of polyphenol intake by classes, OR (95% CI)			
	T1 (Lowest)	T2 (Middle)	T3 (Highest)	*P-*trend ^a^
**Total polyphenols (mg/d)**				
No. of persons, n	167	179	204	
No. of hypertension cases, n (%)	85 (39.0)	77 (23.5)	105 (37.5)	
Dietary intake (mg/d), median (IQR)	190.83 (122.35, 233.01)	360.58 (329.55, 402.15)	566.87 (478.58, 659.72)	
Model 1	1.00	0.42 (0.21, 0.84)	0.77 (0.39, 1.53)	0.470
Model 2	1.00	0.38 (0.19, 0.76)	0.69 (0.34, 1.40)	0.322
Model 3	1.00	0.36 (0.19, 0.69)	0.63 (0.30, 1.31)	0.266
**Phenolic acids (mg/d)**				
No. of persons, n	162	193	195	
No. of hypertension cases, n (%)	96 (34.9)	73 (28.2)	98 (36.9)	
Dietary intake (mg/d), median (IQR)	98.81 (48.07, 167.99)	301.97 (230.39, 344.96)	458.21 (396.59, 632.71)	
Model 1	1.00	0.57 (0.27, 1.20)	0.75 (0.36, 1.54)	0.457
Model 2	1.00	0.47 (0.20, 1.08)	0.65 (0.31, 1.37)	0.284
Model 3	1.00	0.41 (0.18, 0.94)	0.59 (0.28, 1.29)	0.226
**Flavonoids (mg/d)**				
No. of persons, n	178	196	176	
No. of hypertension cases, n (%)	85 (28.3)	100 (39.8)	82 (31.9)	
Dietary intake (mg/d), median (IQR)	31.11 (19.03, 58.06)	34.55 (15.40, 64.78)	41.50 (19.04, 75.07)	
Model 1	1.00	0.86 (0.45, 1.63)	0.90 (0.52, 1.55)	0.702
Model 2	1.00	0.96 (0.52, 1.80)	1.01 (0.53, 1.81)	0.943
Model 3	1.00	0.83 (0.46, 1.51)	0.97 (0.51, 1.84)	0.933
**Tyrosols (mg/d)**				
No. of persons, n	195	200	155	
No. of hypertension cases, n (%)	107 (42.8)	103 (37.2)	57 (20.0)	
Dietary Intake (mg/d), median (IQR)	1.80 (0.93, 3.22)	1.85 (0.97, 2.88)	1.93 (0.98, 3.72)	
Model 1	1.00	0.79 (0.48, 1.28)	0.35 (0.20, 0.61)	<0.001
Model 2	1.00	0.89 (0.52, 1.51)	0.38 (0.21, 0.70)	0.002
Model 3	1.00	0.88 (0.48, 1.59)	0.33 (0.18, 0.64)	0.001
**Alkylphenols (mg/d)**				
No. of persons, n	193	190	167	
No. of hypertension cases, n (%)	102 (41.3)	95 (33.6)	70 (25.1)	
Dietary intake (mg/d), median (IQR)	0.91 (0.41, 1.44)	0.96 (0.49, 1.65)	1.00 (0.50, 1.91)	
Model 1	1.00	0.75 (0.44, 1.29)	0.46 (0.28, 0.76)	0.003
Model 2	1.00	0.77 (0.41, 1.43)	0.46 (0.27, 0.77)	0.005
Model 3	1.00	0.71 (0.39, 1.29)	0.45 (0.23, 0.87)	0.017
**Lignans (mg/d)**				
No. of persons, n	190	199	161	
No. of hypertension cases, n (%)	99 (42.6)	100 (32.6)	68 (24.8)	
Dietary intake (mg/d), median (IQR)	0.09 (0.08, 0.14)	0.10 (0.08, 0.16)	0.11 (0.08, 0.17)	
Model 1	1.00	0.61 (0.35, 1.06)	0.55 (0.30, 1.03)	0.063
Model 2	1.00	0.67 (0.37, 1.21)	0.45 (0.25, 0.83)	0.011
Model 3	1.00	0.63 (0.33, 1.22)	0.49 (0.25, 0.98)	0.048
**Stilbenes (mg/d)**				
No. of persons, n	203	169	178	
No. of hypertension cases, n (%)	111 (41.8)	81 (29.9)	75 (28.3)	
Dietary intake (mg/d), median (IQR)	0.01 (0.00, 0.02)	0.01 (0.00, 0.03)	0.02 (0.01, 0.03)	
Model 1	1.00	0.64 (0.39, 1.07)	0.55 (0.35, 0.86)	0.009
Model 2	1.00	0.72 (0.43, 1.21)	0.67 (0.42, 1.07)	0.093
Model 3	1.00	0.64 (0.35, 1.15)	0.60 (0.36, 0.98)	0.043
**Others (mg/d)**				
No. of persons, n	195	194	161	
No. of hypertension cases, n (%)	108 (41.0)	95 (35.7)	64 (23.3)	
Dietary intake (mg/d), median (IQR)	0.72 (0.37, 1.09)	1.69 (1.43, 1.93)	2.76 (2.22, 3.49)	
Model 1	1.00	0.84 (0.43, 1.64)	0.44 (0.25, 0.75)	0.004
Model 2	1.00	0.89 (0.47, 1.68)	0.46 (0.26, 0.81)	0.009
Model 3	1.00	0.80 (0.41, 1.58)	0.33 (0.14, 0.74)	0.010

Abbreviation: IQR: Interquartile Range, OR: odds ratio. OR and 95% CI were calculated by using multivariate logistic regression. Model 1: Age and sex adjusted. Model 2: Additionally adjusted for race, educational level, body mass index, smoking and physical activity. Model 3: Additionally adjusted for intake of sodium, fiber, saturated fat, alcohol, total energy intake, misreporting, and other polyphenol components. The sample weight was considered for statistical analysis. For this reason, the values of proportion of hypertension cases aren′t compatible with the frequency of the same.

^a^ p-value for trend <0.05 was considered statistically significant.

Logistic regression analyses showed an inverse and significant association, only in the middle tertile, between the total polyphenol intake and phenolic acids with hypertension in all models, such as after multivariate adjustment (OR = 0.36; 95%CI 0.19, 0.69 and OR = 0.41; 95%CI 0.18, 0.94; respectively).

When the analysis was adjusted for potential confounding factors (model 3), an inverse, significant and linearly association between higher intakes of tertiles of tyrosols, as well as alkylphenols, lignans, stilbenes, other polyphenols and hypertension (P-value <0.05), were observed.

Participants in the highest tertile of tyrosols intake had a 67% reduced odds of hypertension (OR = 0.33; 95%CI 0.18, 0.64; P-trend = 0.001). We also found a 55% decrease of hypertension among subjects in the third tertile of alkylphenols (OR = 0.45; 95%CI 0.23, 0.87; P-trend = 0.017) compared to those who were in the first tertile. Furthermore, we observed a reduction in odds of hypertension in patients who consumed more lignans (OR = 0.49; 95%CI 0.25, 0.98; P-trend = 0.048), stilbenes (OR = 0.60; 95%CI 0.36, 0.98; P-trend = 0.043), and other polyphenols (OR = 0.33; 95%CI 0.14, 0.74; P-trend = 0.010). However, total flavonoid intake was not significantly associated with hypertension (P-trend = 0.933).

## Discussion

The present study reports significant and inverse associations between higher polyphenol intakes, such as lignans, stilbenes, tyrosols, alkylphenols, and other polyphenols and hypertension in a population based-study.

Experimental and observational studies support the argument that a polyphenol-rich diet may have a beneficial effect on BP, helping to lower high BP and prevent it from increasing, by different mechanisms and, in some cases, different polyphenolic structures [2, 9, 29]. Thereby, it has been demonstrated, *in vivo*, that polyphenols and their metabolites have anti-atherosclerotic effects, improve endothelial function and exert antioxidant activities by inhibit enzimes generating reactive oxygen species (ROS) as xanthine oxidase and nicotinamide adenine dinucleotide phosphate (NADPH) oxidase. Indirectly, polyphenols can interfere with the cellular detoxification systems, such as superoxide dismutase, catalase or glutathione peroxidases. What's more, they present anti-inflammatory effects, increase nitric oxide release, and modulate inflammation and lipid metabolism, leading to lower low-density lipoprotein (LDL) oxidation and platelet aggregation [2, 3, 30]. However, in the present study, the total polyphenol intake in the highest tertile was not significantly associated with lower odds of hypertension. It can be explained due to lower intake of total polyphenols comparing with the findings reported by Tresserra-Rimbau *et al*. in the Spanish population [30]. This difference can be explained by the individual food preferences and the difference in dietary habits among both populations that are often dictated by culture. Thus, the food pattern can affect the intake of classes and the amount of polyphenols. Besides, coffee is the first contributor of polyphenol intake in the diet of our population. In addition, despite the presence of antioxidants and other bioactive compounds in coffee, it is the main source of caffeine, a stimulant and psychoactive substance [31]. According to a recent review, caffeine intake is associated with an increase in BP, systemic vascular resistance, arterial stiffness and it has unfavorable effects on endothelial function in healthy subjects [31].

Similarly, phenolic acids were not significantly associated with the decrease of hypertension in the highest tertile. This can be explained because the main polyphenol class was phenolic acids and coffee was also the primary food item contributing to phenolic acids intake.

By contrast, within the other polyphenol classes, our results indicate a potential role of lignans in reducing hypertension. Although sesame seed oil is the main source of this polyphenol group in our population, lignans may be consumed in higher amounts through flax, sesame seeds and olive oil. This finding can be corroborated by another study among the Spanish population [30]. A double-blind, cross-over, placebo-controlled trial study has revealed that 4-weeks administration of sesamin, one of the major lignans derived from sesame seeds, has antihypertensive properties in humans [32].

The reported inverse association, specifically for red wine and olive oil [33, 34], is consistent with the inverse and significant association we found for stilbenes and tyrosols, respectively, with hypertension. In our population, stilbenes intake came mostly from red wine and berries, mainly grapes and strawberries. The most known compound of this family of molecules is resveratrol. As it is abundantly available in grape skins (predominantly as resveratrol-3-Obeta-gluco-side) and conserved after wine elaboration, red wine represents its main source in the human diet [29]. Thereby, it is apparent that polyphenols from red wine reduce BP elevations and improve structural and functional cardiovascular alterations caused by chronic inhibition of nitric oxide synthase, consequently, they attenuate end-organ damage such as myocardial fibrosis and aortic thickening, and decrease protein synthesis in the heart and aorta [2, 33].

In this study, olives and olive oil were the main food contributors to tyrosol intake. It was described that olive oil can exert beneficial effects on cardiovascular risk factors by diminishing BP, regulating plasma lipid levels, reducing systemic inflammation and repairing oxidative damage [34, 35]. This is probably due to the high amount of monounsaturated fatty acids, but it is likely to be also the result of the presence of biophenols, which have a strong antioxidant power [35]. A recent study claimed that polyphenols from olives enhance NO concentrations and may help dilate arteries, stimulate ROS synthase system, and increase plasma nitrites/nitrates, which reduces BP [36]. In our study, the highest tertile of tyrosols was associated with a 66% reduced odds of hypertension. De la Torre (2010) [34] stated that a low concentration of tyrosols might be enough to exert their antioxidant activities and the study of Di Benedetto *et al*. [35] suggest that tyrosol is effective in preserving cellular antioxidant defense, protecting Caco-2 cells against the cytotoxic/ apoptotic effects of oxidized-LDL and inhibiting the activity of the leukocyte 5-lipoxygenase.

We also found an inverse association between the alkylphenols intake and hypertension. However, the cardioprotective effect of this class of polyphenols was not previously reported in another study. Alkylphenols are mostly present in whole grain bread and whole grain flour. Jacobs *et al*. [37] have suggested that an increased intake of whole grains may protect against coronary heart diseases and may have beneficial effects on lowering the risk of cardiovascular disease.

We also observed a trend toward a reduction of hypertension with the increasing intake of other polyphenols; a heterogeneous group. Despite the largest contributor being the coffee, this presents smaller amounts when compared to the total polyphenols and phenolic acids. Also, in this group there are different food contributors, such as, olives and olive oil, well known for decrease BP, as mentioned previously.

Epidemiological data of dietary flavonoids and beneficial cardiovascular effects in human populations have been inconsistent. Some prospective studies have reported statistically significant inverse association between the total flavonoid intake and cardiovascular disease incidence or mortality [38, 39]. But other prospective study has not found similar results [40]. In the present study, we found no association between flavonoids intake and hypertension. That can be explained because in our research the flavonoids intake of 45.6 (SE = 2.8) mg/day was lower than what was reported in other studies [30, 39].

Furthermore, the results of this study should be interpreted based on some limitations. First, our study is its cross-sectional nature, which does not allow definitive establishment of causal inference. Additionally, there are clinical conditions that affect hypertension and dietary intake, such as diabetes, CVD, kidney disease, sleep apnea, stoke and cancer. However, the prevalence of these diseases could not be considered in the statistical models because we do not have the diagnostic information of these diseases by the participants, but only the referred information, which was not validated. Although the Phenol-Explorer is the most complete database available currently, the information about some regional foods consumed in Brazil is still scarce because they have not been characterized or only poorly characterized (e.g. cassava flour, tapioca, sweet potato, coconut, and coconut milk). However, despite the database does not contemplate information of these foods, their consumption were low or non-existent. Moreover, similarly to other studies [16, 30], we can demonstrate that though the median dietary intakes of these polyphenol classes (e.g. tyrosols, alkylphenols, lignans, stilbenes, other polyphenols) were lower compared with other groups (e.g. phenolic acids and flavonoids) it is possible to observe a beneficial effect of their actions in human health so, their effects can not be disregarded despite the low amounts. Nevertheless, additional epidemiological studies are needed to definitively clarify the benefits deriving from long-term consumption of these classes of polyphenols in cardiovascular health. Also, further investigation in large prospective studies of potential cardioprotective effects of the intake of polyphenols and in intervention studies designed to test optimal doses of polyphenol-rich foods to establish future daily polyphenol intake recommendations for the prevention of hypertension.

## Conclusions

Data from the present study allowed us to conclude that there is an inverse and significant association between some classes of polyphenols, such as, tyrosols, alkylphenols, lignans, stilbenes, other polyphenols and hypertension, showing the importance of the consumption of polyphenol-rich foods. Thus, these results may be useful to identify specific food sources of polyphenols that may reduce the odds of hypertension. However, further clinical trials are needed to confirm the promising protective effects of polyphenols on hypertension and establish desired minimum levels of intake.

## Supporting Information

S1 Fig Sample of ISA-Capital study. Sao Paulo, Brazil.
(TIF)

## References

[1] Manach C, Mazur A, Scalbert A. Polyphenols and prevention of cardiovascular disease. Curr Opin Lipidol. 2005;16: 77–84. 15650567 10.1097/00041433-200502000-00013

[2] Andriantsitohaina R, Auger C, Chataigneau T, Étienne-Selloum N, Li H, Martínez MC, et al Molecular mechanisms of the cardiovascular protective effects of polyphenols. Br J Nutr. 2012;108: 1532–1549. 10.1017/S0007114512003406 22935143

[3] Manach C, Scalbert A, Morand C, Rémésy C, Jimenez L. Polyphenols: food sources and bioavailability. Am J Clin Nutr. 2004;79: 727–747. 15113710 10.1093/ajcn/79.5.727

[4] Tsao R. Chemistry and Biochemistry of Dietary Polyphenols. Nutrients. 2010;2: 1231–1246. 10.3390/nu2121231 22254006 PMC3257627

[5] Pérez-Jiménez J, Neveu V, Vos F, Scalbert A. Systematic Analysis of the Content of 502 Polyphenols in 452 Foods and Beverages: An Application of the Phenol-Explorer Database. J Agric Food Chem. 2010;58: 4959–4969. 10.1021/jf100128b 20302342

[6] Stoclet JC, Chataigneau T, Ndiaye M, Oak MH, El Bedoui J, Chataigneau M, et al Vascular protection by dietary polyphenols. Eur J Pharmacol500. 2004;500: 299–313. 10.1016/j.ejphar.2004.07.034 15464042

[7] Wang Y, Chun OK, Song WO. Plasma and Dietary Antioxidant Status as Cardiovascular Disease Risk Factors: A Review of Human Studies. Nutrients. 2013;5: 2969–3004. 10.3390/nu5082969 23912327 PMC3775238

[8] Sociedade Brasileira de Cardiologia, Sociedade Brasileira de Hipertensão, Sociedade Brasileira de Nefrologia. V Diretrizes brasileiras de hipertensão arterial. Arq Bras Cardiol. 2007;89: e24–e79. 17906811

[9] Medina-Remón A, Estruch R, Tresserra-Rimbau A, Vallverdú-Queralt A, Lamuela-Raventos RM. The effects of polyphenol consumption on blood pressure. Mini Rev Med Chem. 2013;13: 1137–1149. 22931531 10.2174/1389557511313080002

[10] ESH/ESC Task Force for the Management of Arterial Hypertension. Guidelines for the Management of Arterial Hypertension: The Task Force for the Management of Arterial Hypertension of the European Society of Hypertension and of the European Society of Cardiology. J Hypertens. 2013;31: 1281–1357. 10.1097/01.hjh.0000431740.32696.cc 23817082

[11] Chobanian AV. The hypertension paradox-More uncontrolled disease despite improved therapy. N Engl J Med. 2009;361: 878–887. 10.1056/NEJMsa0903829 19710486

[12] Appel LJ, Brands MW, Daniels SR, Karanja N, Elmer PJ, Sacks FM; American Heart Association. Dietary approaches to prevent and treat hypertension: a scientific statement from the American Heart Association. Hypertension. 2006;47: 296–308. 10.1161/01.HYP.0000202568.01167.B6 16434724

[13] Utsugi MT, Ohkubo T, Kikuya M, Kurimoto A, Sato RI, Suzuki K, et al Fruit and Vegetable Consumption and the Risk of Hypertension Determined by Self Measurement of Blood Pressure at Home: The Ohasama Study. Hypertens Res. 2008;31: 1435–1443. 10.1291/hypres.31.1435 18957815

[14] Nakachi K, Matsuyama S, Miyake S, Suganuma M, Imai K. Preventive effects of drinking green tea on cancer and cardiovascular disease: epidemiological evidence for multiple targeting prevention. Bio Factors. 2000;13: 49–54. 10.1002/biof.5520130109 11237198

[15] Hooper L, Kay C, Abdelhamid A, Kroon PA, Cohn JS, Rimm EB, et al Effects of chocolate, cocoa, and flavan-3-ols on cardiovascular health: a systematic review and meta-analysis of randomized trials. Am J Clin Nutr. 2012;95: 740–751. 10.3945/ajcn.111.023457 22301923

[16] Tresserra-Rimbau A, Rimm EB, Medina-Remón A, Martínez-González MA, López-Sabater MC, Covas MI, et al Polyphenol intake and mortality risk: a re-analysis of the PREDIMED trial. BMC Medicine. 2014;12: 77 10.1186/1741-7015-12-77 24886552 PMC4102266

[17] Medina-Remón A, Tresserra-Rimbau A, Pons A, Tur JA, Martorell M, Ros E, et al Effects of total dietary polyphenols on plasma nitric oxide and blood pressure in a high cardiovascular risk cohort. The PREDIMED randomized trial. Nutr Metab Cardiovasc Dis. 2015;25: 60–67. 10.1016/j.numecd.2014.09.001 25315667

[18] Instituto Brasileiro de Geografia e Estatística. Pesquisa Nacional por Amostra de Domicílios (2005) [Government Reseach]. Available: http://www.ibge.gov.br/home/estatistica/populacao/trabalhoerendimento/pnad2005/.

[19] Guenther PM, DeMaio TJ, Ingwersen LA, Berline M. The multiple-pass approach for the 24 hour recall in the Continuing Survey of Food Intakes by Individuals (CSFII) 1994–1996. Paper presented at the International Conference on Dietary Assessment Methods. Boston, Mass; 1995.

[20] Blanton CA, Moshfegh AJ, Baer DJ, Kretsch MJ. The USDA Automated Multiple-Pass Method accurately estimates group total energy and nutrient intake. J Nutr. 2006;136: 2594–2599. 16988132 10.1093/jn/136.10.2594

[21] NDSR. Nutrition Data System for Research. Minneapolis: University of de Minnesota; 2005.

[22] Haubrock J, Nöthlings U, Volatier JL, Dekkers A, Ocke M, Harttig U, et al Estimating usual food intake distributions by using the multiple source method in the EPIC-Potsdam Calibration Study. J Nutr. 2011;141: 914–920. 10.3945/jn.109.120394 21430241

[23] Mendez MA, Popkin BM, Buckland G, Schroder H, Amiano P, Barricarte A, et al Alternative Methods of Accounting for Underreporting and Overreporting When Measuring Dietary Intake-Obesity Relations. Am J Epidemiol. 2011;173: 448–458. 10.1093/aje/kwq380 21242302 PMC3139974

[24] Black AE. Critical evaluation of energy intake using the Goldberg cut-off for energy intake: basal metabolic rate. A practical guide to its calculation, use and limitations. Int J Obes Relat Metab Disord. 2000;24: 1119–1130. 11033980 10.1038/sj.ijo.0801376

[25] Miranda AM, Steluti J, Fisberg RM, Marchioni DM. Dietary intake and food contributors of polyphenols in adults and elderly adults of Sao Paulo: a population-based study. Br J Nutr 2016;115: 1061–1070. 10.1017/S0007114515005061 26810764

[26] World Health Organization. Obesity: preventing and managing the global epidemic Report of a World Health Organization Consultation. Geneva: World Health Organization WHO Obesity Technical Report Series; 2000. 11234459

[27] Craig CL, Marshall AL, Sjostrom M, Bauman AE, Booth ML, Ainsworth BE, et al International physical activity questionnaire: 12-country reliability and validity. Med Sci Sports Exerc. 2003;35: 1381–1395. 10.1249/01.MSS.0000078924.61453.FB 12900694

[28] Matsudo S, Araujo T, Matsudo V, Andrade D, Andrade E, Oliveira LC, et al Questionário internacional de atividade física (IPAQ): estudo de validade e reprodutibilidade no brasil. Rev Bras Ativ Fís Saúde. 2001;6: 5–12.

[29] Galleano M, Pechanova O, Fraga CG. Hypertension, Nitric Oxide, Oxidants, and Dietary Plant Polyphenols. Curr Pharm Biotechnol. 2010;11: 837–848. 20874688 10.2174/138920110793262114

[30] Tresserra-Rimbau A, Rimm EB, Medina-Remón A, Martínez-González MA, de la Torre R, Corella D, et al Inverse association between habitual polyphenol intake and incidence of cardiovascular events in the PREDIMED study. Nutr Metabol Cardiovasc Dis. 2014;24: 639–647. 10.1016/j.numecd.2013.12.014 24552647

[31] Di Castelnuovo A, Giuseppe R, Iacoviello L, de Gaetano G. Consumption of cocoa, tea and coffee and risk of cardiovascular disease. Eur J Intern Med. 2012;23: 5–25. 10.1016/j.ejim.2011.07.014 22153525

[32] Miyawaki T, Aono H, Toyoda-Ono Y, Maeda H, Kiso Y, Moriyama K. Antyhipertensive effects of sesamin in humans. J Nutr Sci Vitaminol. 2009;55: 87–91. 19352068 10.3177/jnsv.55.87

[33] Bernátová I, Pechánová O, Babál P, Stvrtina S, Andriantsitohaina R. Wine polyphenols improve cardiovascular remodeling and vascular function in NO-deficient hypertension. Am J Physiol Heart Circ Physiol. 2002;282: 942–948. 10.1152/ajpheart.00724.2001 11834490

[34] de la Torre-Carbot K, Chávez-Servín JL, Castellote AI, Lamuela-Raventós RM, Nurmi T, Poulsen HE, et al Elevated Circulating LDL Phenol Levels in Men Who Consumed Virgin Rather Than Refined Olive Oil Are Associated with Less Oxidation of Plasma LDL. J Nutr. 2010;140: 501–508. 10.3945/jn.109.112912 20089783

[35] Di Benedetto R, Vari R, Scazzocchio B, Filesi C, Santangelo C, Giovannini C, et al Tyrosol, the major extra virgin olive oil compound, restored intracellular antioxidant defences in spite of its weak antioxidative effectiveness. Nutr Metab Cardiovasc Dis. 2007;17:535–545. 10.1016/j.numecd.2006.03.005 16928436

[36] Moreno-Luna R, Muñoz-Hernandez R, Miranda ML, Costa AF, Jimenez-Jimenez L, Vallejo-Vaz AJ, et al Olive Oil Polyphenols Decrease Blood Pressure and Improve Endothelial Function in Young Women with Mild Hypertension. Am J Hypertens. 2012;25: 1299–1304. 10.1038/ajh.2012.128 22914255

[37] Jacobs Jr, Meyer KA, Kushi LH, Folsom AR. Whole-grain intake may reduce the risk of ischemic heart disease death in postmenopausal women: the Iowa Women’s Health Study. Am J Clin Nutr. 1998;68: 248–257. 9701180 10.1093/ajcn/68.2.248

[38] Knekt P, Jarvinen R, Reunanen A, Maatela J. Flavonoid intake and coronary mortality in Finland: a cohort study. BMJ. 1996;312: 478–481. 8597679 10.1136/bmj.312.7029.478 PMC2349921

[39] McCullough ML, Peterson JJ, Patel R, Jacques PF, Shah R, Dwyer JT. Flavonoid intake and cardiovascular disease mortality in a prospective cohort of US adults. Am J Clin Nutr. 2012;95: 454–464. 10.3945/ajcn.111.016634 22218162 PMC3260072

[40] Lin J, Rexrode KM, Hu F, Albert CM, Chae CU, Rimm EB, et al Dietary intakes of flavonols and flavones and coronary heart disease in US women. Am J Epidemiol. 2007;165: 1305–1313. 10.1093/aje/kwm016 17379619

