# Supplementary figures and images for: Association between Polyphenol Intake and Hypertension in Adults and Older Adults: A Population-Based Study in Brazil
Source: PLoS One. 2016 Oct 28;11(10):e0165791. doi: 10.1371/journal.pone.0165791 (PMC5085083; doi:10.1371/journal.pone.0165791)

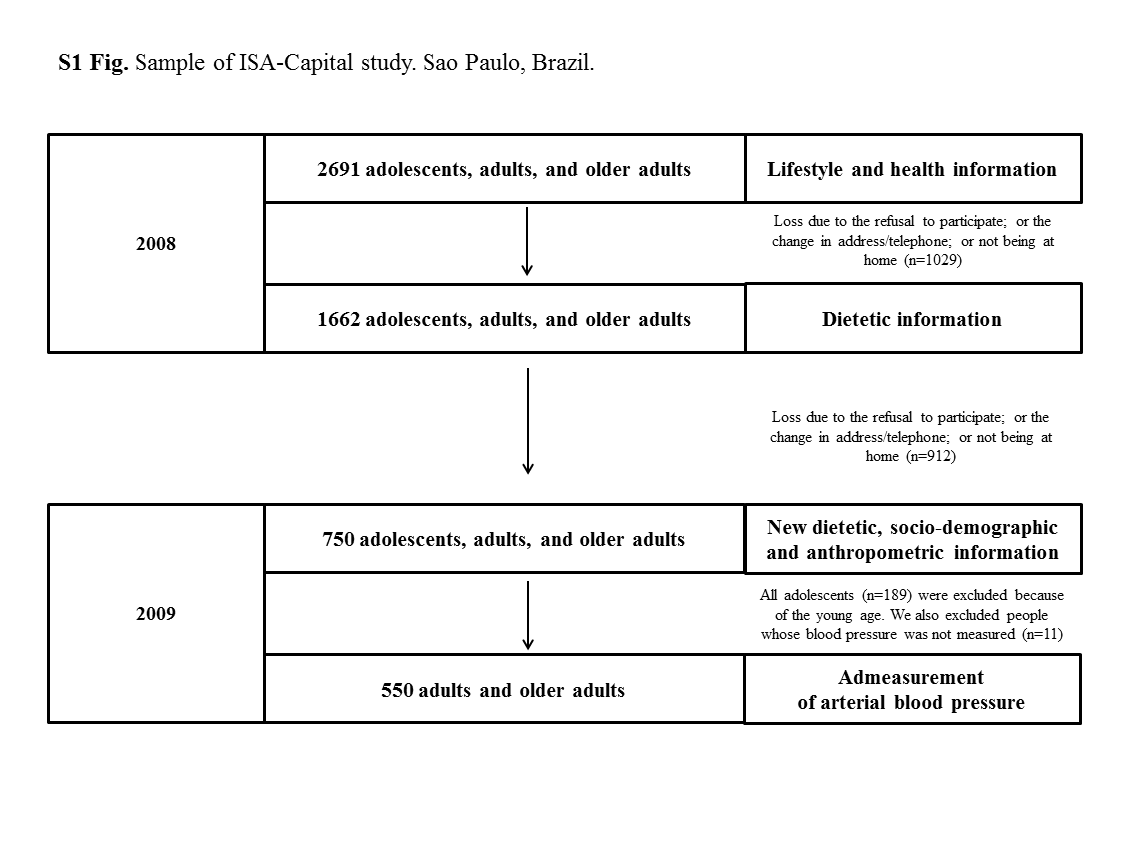

Supplement: S1 Fig — Sao Paulo, Brazil. (TIF) [file pone.0165791.s001.tif]
